# Sabiporide Improves Cardiovascular Function, Decreases the Inflammatory Response and Reduces Mortality in Acute Metabolic Acidosis in Pigs

**DOI:** 10.1371/journal.pone.0053932

**Published:** 2013-01-10

**Authors:** Dongmei Wu, Jeffrey A. Kraut, William M. Abraham

**Affiliations:** 1 Department of Research, Division of Neonatology, Mount Sinai Medical Center, Miami Beach, Florida, United States of America; 2 Division of Pulmonary and Critical Care Medicine, Mount Sinai Medical Center, Miami Beach, Florida, United States of America; 3 Department of BIN Fusion Technology, Chonbuk National University, Jeonju, Korea; 4 Division of Nephrology, Medical and Research Services Veterans Administration Greater Los Angeles Healthcare System and David Geffen School of Medicine, Los Angeles, California, United States of America; University of Leicester, United Kingdom

## Abstract

**Introduction:**

Acute metabolic acidosis impairs cardiovascular function and increases the mortality of critically ill patients. However, the precise mechanism(s) underlying these effects remain unclear. We hypothesized that targeting pH-regulatory protein, Na^+^/H^+^ exchanger (NHE1) could be a novel approach for the treatment of acute metabolic acidosis. The aim of the present study was to examine the impact of a novel NHE1 inhibitor, sabiporide, on cardiovascular function, blood oxygen transportation, and inflammatory response in an experimental model of metabolic acidosis produced by hemorrhage-induced hypovolemia followed by an infusion of lactic acid.

**Methods and Results:**

Anesthetized pigs were subjected to hypovolemia for 30 minutes. The animals then received a bolus infusion of sabiporide (3 mg/kg) or vehicle, followed by an infusion of lactic acid for 2 hours. The animals were continuously monitored for additional 3 hours. Hypovolemia followed by a lactic acid infusion resulted in a severe metabolic acidosis with blood pH falling to 6.8. In association with production of the acidemia, there was an excessive increase in pulmonary artery pressure (PAP) and pulmonary vascular resistance (PVR). Treatment with sabiporide significantly attenuated the increase in PAP by 38% and PVR by 67%, as well as significantly improved cardiac output by 51%. Sabiporide treatment also improved mixed venous blood oxygen saturation (55% in sabiporide group vs. 28% in control group), and improved systemic blood oxygen delivery by 36%. In addition, sabiporide treatment reduced plasma levels of TNF-α (by 33%), IL-6 (by 63%), troponin-I (by 54%), ALT (by 34%), AST (by 35%), and urea (by 40%).

**Conclusion:**

These findings support the possible beneficial effects of sabiporide in the treatment of acute metabolic acidosis and could have implications for the treatment of metabolic acidosis in man.

## Introduction

Acute metabolic acidosis developing with sepsis, cardiogenic shock, and hemorrhagic shock contributes to the high mortality of these disorders [1]. Indeed, the severity of the metabolic acidosis, as reflected by arterial blood pH, has been found in several studies to be an important predictor of clinical outcome i.e., the more severe the acidemia the greater the mortality [1], [2]. The acidosis contributes to an increase in morbidity and mortality by impairing the function of several organ systems, but primarily the cardiovascular system [3]. In this regard, acidosis is associated with depression of cardiac contractility, increased susceptibility to cardiac arrhythmias, and increased pulmonary vascular resistance, with a predisposition to hypotension and decreased tissue perfusion [2]–[6]. In addition to its impact on the cardiovascular system, experimental studies have revealed evidence of suppression of the immune response and stimulation of an inflammatory state [7], [8].

The detrimental effects of metabolic acidosis have been attributed to changes in critical protein functions arising from alterations in extracellular and intracellular pH [9]. Therefore, emphasis has been placed on amelioration of the acidosis and acidemia by administration of base. However, base therapy has not resulted in a consistent reduction in mortality [10]–[13]. Studies from our laboratory and that of others have established that the intracellular acidosis present with acute metabolic acidosis causes activation of the Na^+^/H^+^ exchanger (NHE1), a ubiquitous plasma-membrane transport system that functions in the regulation of cytoplasmic pH, resulting in deleterious increments in intracellular sodium and calcium and impairment in cardiovascular function [14]–[17]. Consistent with this theory are findings that administration of a selective NHE1 inhibitor to animals with hemorrhagic shock or sepsis attenuates the depression in cardiovascular function and leads to a decrease in mortality [17], [18]. Using a porcine model of asphyxia-induced cardiac arrest, a model in which hypoxia of tissues and the resultant lactic acidosis is more global, we recently showed that administration of sabiporide, a potent and selective NHE1 inhibitor affords protection from whole body ischemia-reperfusion injury by attenuating myocardial dysfunction, improving organ blood flows and systemic oxygen delivery, resulting in reduced pro-inflammatory response [19]. These findings suggest that administration of selective inhibitors of NHE1 lessen the extent of cellular injury, and improve survival. However, in those animal models of hypovolemic circulatory shock, fluid resuscitation results in a normalization of systemic pH. It is not clear whether NHE1 inhibitors can afford protection from severe and persistent systemic metabolic acidosis that seen with critically ill patients. In the present study, we examined the impact of administration of sabiporide on cardiovascular and metabolic function, proinflammatory cytokine production in a model of acute metabolic acidosis produced by hemorrhagic hypotension followed by a lactic acid infusion to produce severe systemic metabolic acidosis [20], [21]. The results of these studies demonstrate that administration of sabiporide improves cardiovascular and metabolic function, and attenuates proinflammatory cytokine production. Moreover, this treatment reduces mortality, despite having little impact on the severity of the metabolic acidosis. These studies give further support to the possible beneficial effects of administration of NHE1 inhibitors in the treatment of acute metabolic acidosis.

## Materials and Methods

### Animal Preparation

All animal studies were approved by the Institutional Animal Care and Use Committee at Mount Sinai Medical Center of Florida and complied with the Animal Welfare Act. Fourteen male Yorkshire pigs (29.4±4.5 kg) were anesthetized with ketamine, 10 mg/kg, i.m., and maintained in a surgical plane of anesthesia with intravenous propofol. A cuffed endotracheal tube was placed through the oropharynx, and ventilation was provided on room air with a volume-controlled ventilator (PB 7200 Ventilatory System, Puritan-Bennett, Carlsbad, CA) set to deliver a tidal volume of 10 ml/kg. The respiratory rate was adjusted to ensure a P_a_CO_2_ from 35–45 mmHg. The left external jugular vein was cannulated for the administration of fluids and drugs. Another catheter was placed into the right femoral artery for the measurement of arterial blood pressure and for blood sampling. An 5.5 F balloon-tipped flow directed thermodilution pulmonary arterial catheter (Opticom™, Abbot Laboratories, Chicago, IL, USA) was inserted via the right jugular vein and floated into the pulmonary artery under direct pressure monitoring for measurements of pulmonary arterial pressure, right atrial pressure, core body temperature and cardiac output. All hemodynamic parameters were continuously recorded with a Powerlab data acquisition system. Arterial and central venous blood gases were measured at various intervals during the experiments using a blood gas analyzer (Rapidlab 855, Bayer Corporation, New York). The cardiac output was determined by thermodilution in triplicate using ice-cold saline. Body temperature was maintained between 37°C to 39°C by means of heating pad.

### Experimental Protocol

**Figure 1:**
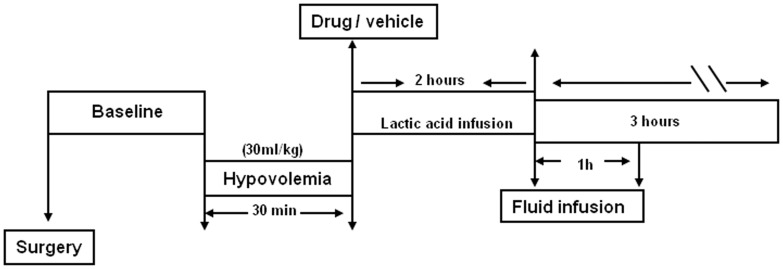
Details of the experimental protocol are shown. Anesthetized pigs were subjected to hypovolemia (30 ml/kg) for 30 minutes. The animals then received a bolus infusion of NHE1 inhibitor (3 mg/kg, sabiporide) or vehicle, followed by lactic acid infusion for 2 hours. The animals were continuously monitored for additional 3 hours.

The experimental protocol utilized is depicted in Figure 1. Following completion of the surgical procedure, the animals were allowed to stabilize for 30 minutes. Hypovolemia was then induced by bleeding the pigs through a femoral artery catheter (30 ml/kg blood removed within 30 minutes). The animals were then randomly assigned to receive either 3 mg/kg sabiporide (in 25 ml saline) or vehicle (saline). Personnel performing the studies were unaware of the assignment of the groups. All animals were given an infusion of lactic acid for 2 hours (1 M lactic acid in saline, 20 ml/kg/h for the first 30 min, 10 ml/kg/h for 90 min), followed by 10 ml/kg of Lactated Ringer’s administered for an additional 60 minutes. The animals were monitored throughout the procedure and for an additional 2 hours. At the end of experiments, the animals were humanely euthanized while still under anesthesia with 10 ml of Euthanol, a method that is consistent with the recommendation of the Panel on Euthanasia of the American Veterinary Medical Association.

### Echocardiography

Echocardiography was performed with the use of a Hewlett-Packard echocardiographic system SONOS 2000 with a 3.5/2.7 MHz transducer and recorded on VCR tapes. In each animal two-dimensional short-axis view was taken at mid-papillary muscle level to obtain left ventricular (LV) ejection fraction (EF). Linear dimensions were measured from two-dimensionally guided M-mode tracing, and fractional shortening (FS) was obtained. An ECG tracing was recorded simultaneously with the echocardiogram. The measurements were made after the recommendation of the American Society of Echocardiography [22], [23]. Wall motion score index (WMSI) was obtained by the sum of wall motion scores divided the number of visualized segments. In this scoring system, higher scores indicate more severe wall motions abnormalities as: 1 = normal, 2 = hypo kinesis, 3 = akinesis, 4 = dyskinesis, 5 = aneurysm [22]. The measurements were made both on line and off line. All measurements were repeated three times, and the results were averaged.

### Biochemical Assay

Levels of tumor necrosis factor (TNF)-α, IL-6 were determined by using enzyme immunoassay kits according to the manufacturer’s instructions (R&D Systems, Minneapolis, MN, USA). Plasma Troponin-I levels were measured by using a pig cardiac troponin-I enzyme immunoassay kit (Life Diagnostics, Inc., West Cheater, PA). Plasma levels of alanine aminotransferase (ALT) and aspartate aminotransferase (AST) (Biotron Diagnostics, Hemet, CA), and plasma levels of urea (Bioassay System, Hayward, CA) were determined by using assay kits according to the manufacturer’s instructions.

### Statistical Analysis

All data were reported as means ± SD. Statistical analysis was performed by one way ANOVA and Bonferroni’s post comparison test for repeated measures. P values <0.05 were considered to be statistically significant.

## Results

**Figure 2:**
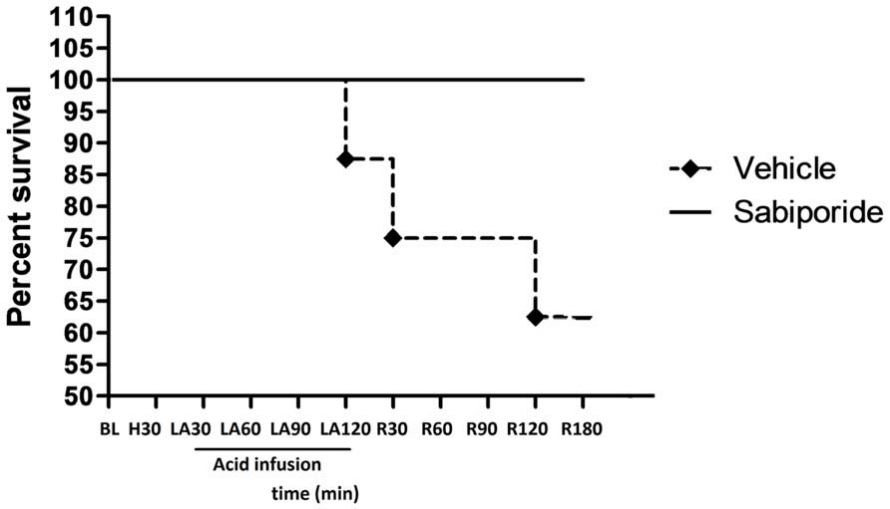
Survival of animals. Three of the eight control animals died either during infusion of lactic acid or after its discontinuation. By contrast, all six animals given sabiporide survived. *P = 0.0002 vs. the control group by chi square analysis for trend. BL: at baseline; H30: at 30 min of hemorrhage; T30 min, –T300 min: at 30 min, –and 300 min from lactic acid infusion.

Hypovolemia followed by lactic acid infusion led to the early death of 3 of the 8 control animals (38%). These three animals were died due to ventricular fibrillation-induced sudden cardiac arrest. In contrast, all of the six animals given the sabiporide survived the experimental protocol (Fig. 2).

### Acid-base Parameters

Acid-base parameters in controls and animals given sabiporide are shown in Table 1. Baseline arterial blood pH, 7.47±.02 and arterial blood [HCO_3_^−^], 24±0.8 mEq/l in controls were not different from that in animals given sabiporide 7.48±.02 and 25±0.5 mEq/l, respectively. Hypotension followed by infusion of lactic acid caused plasma bicarbonate to fall by approximately 16 mEq/l in both groups, and resulting in a blood pH which averaged 6.85±0.05 and 6.87±0.06 in controls and animals given sabiporide, respectively.

**Table 1:** Changes in acid-base parameters and blood oxygenation after hypovolemia and lactic acidosis.

Parameters	Groups	BL	H30 min	T30 min	T60 min	T120 min	T180 min	T240 min	T300 min
**Arterial pH**	control	7.47±0.02	7.46±0.01	6.85±0.05#	6.98±0.03#	6.92±0.03#	7.23±0.06#	7.39±0.03	7.45±0.06
	sabiporide	7.48±0.02	7.48±0.03	6.87±0.06#	7.00±0.06#	6.97±0.07#	7.27±0.06#	7.33±0.08#	7.38±0.06
**Arterial pCO2**	control	34.1±1.4	35.2±1.3	51.8±5.5#	41.1±1.1#	42.8±4.0#	30.0±4.1	33.9±2.4	31.8±4.7
(mmHg)	sabiporide	34.7±1.4	35.4±2.1	49.4±2.8#	41.2±3.3#	39.6±1.7#	34.1±1.7	36.6±1.7	36.3±1.0
**Arterial pO2**	control	107±4	102±6	107±8	104±6	92±12#	95±5	99±3	101±5
(mmHg)	sabiporide	106±8	105±8	104±5	112±9	98±9	104±5	94±8	94±7
**Arterial bicarbonate**	control	24.1±0.8	24.3±0.5	8.8±0.8#	9.5±0.6#	8.5±0.4#	13.1±2.8#	20.1±0.2#	21.4±0.9#
(mM/L)	sabiporide	25.4±0.5	25.6±0.3	9.0±1.0#	10.2±1.3#	9.1±1.1#	15.7±2.5#	19.4±2.5#	21.5±2.6#
**ctHb**	control	10.1±0.4	9.7±0.3	8.1±0.2#	7.2±0.1#	6.9±0.2#	6.7±0.3#	7.1±0.5#	6.9±0.4#
(g/dL)	sabiporide	9.8±0.2	9.9±0.1	7.9±0.6#	7.6±0.4#	7.4±0.4#	6.7±0.6#	7.6±0.2#	7.5±0.1#
**Hematocrit**	control	29.6±1.1	29.0±0.9	24.0±0.4#	21.0±0.4#	20.3±0.8#	19.5±0.6#	21.0±1.2#	20.0±1.0#
(%)	sabiporide	28.8±0.6	29.5±0.3	23.3±1.7#	22.3±1.3#	21.8±1.1#	19.5±1.7#	22.3±0.6#	22.0±0.8#
**Arterial O2 content**	control	14.1±0.5	13.6±0.4	10.5±0.4#	9.6±0.1#	8.4±0.8#	8.9±0.5#	9.7±0.6#	9.6±0.5#
(ml/dL)	sabiporide	13.3±0.5	13.3±0.8	10.6±0.8#	10.1±0.5#	9.6±0.8# *	9.2±0.7#	10.0±0.6#	9.7±0.6#
**Mix-V O2 content**	control	9.6±0.4	4.9±0.1#	5.9±0.2#	5.4±0.2#	3.2±0.6#	3.3±0.5#	3.4±0.4#	3.2±0.4#
(ml/dL)	sabiporide	9.1±0.6	5.2±1.2#	6.2±0.9#	5.7±0.4#	5.4±0.6# *	4.5±0.7# *	4.6±0.7# *	4.6±0.4# *
**Oxygen consumption**	control	4.4±0.3	3.3±0.3#	4.6±0.5	4.8±0.5	4.9±0.4	5.4±0.5	5.0±0.4	4.4±0.2
(ml/kg/min)	sabiporide	4.5±0.5	3.2±0.6#	4.1±0.3	3.9±0.3*	4.8±0.9	5.0±0.2	6.1±0.4# *	5.6±0.5# *

All values are expressed as the mean ± SD (n = 5–6).

* p<0.05 vs. the control group;

# p<0.05 vs. the baseline value. BL: baseline; H30: at 30 minutes of hypovolemia; T30 min, –T300 min: at 30 min, –and 300 min from lactic acid infusion.

### Effects of Sabiporide on Hemodynamic Parameters and Cardiac Function

**Figure 3:**
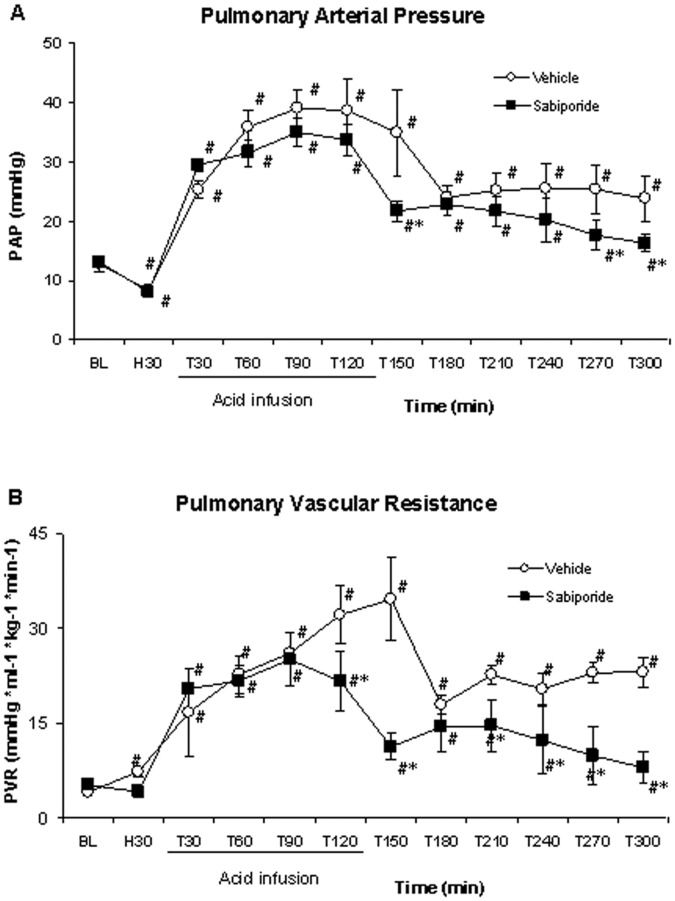
Changes in pulmonary arterial pressure and pulmonary vascular resistance. Pulmonary arterial pressure and vascular resistance rose in controls with lactic acid infusions and remained elevated throughout the study. The increase in both parameters was blunted with sabiporide. All values are the mean ± SD. N = 5–6. *p<0.05 vs. the control group; #p<0.05 vs. the baseline value.

Hemorrhage alone resulted in severe depression of cardiac index and a fall in blood pressure in both groups (Table 2): Cardiac output decreased by approximately 61%, and mean arterial blood pressure (MAP) decreased by approximately 65% in both groups within the first 30 min following hemorrhage. The subsequent infusion of lactic acid to controls caused an increase in pulmonary artery pressure (PAP, ↑ by 205%), pulmonary vascular resistance (PVR, ↑ by 746%), and right atrial pressure (RAP, ↑ by 232%). There was an initial improvement in arterial blood pressure and cardiac output, presumably due, in part, to volume expansion caused by the fluid administration. However, the cardiac output subsequently decreased reaching values significantly below baseline. In the group receiving sabiporide, the infusion of lactic acid also caused an initial rise in cardiac output. However, in contrast to the control group, sabopiride administration caused cardiac output to remain elevated at values that were significantly higher than control and close to baseline. Moreover, the increments in pulmonary arterial pressure, pulmonary vascular resistance and right atrial pressure observed in the control group were considerably dampened, resulting in a reduced preload and improved cardiac output (Figure 3).

**Table 2:** Changes in hemodynamics after hypovolemia and lactic acidosis in pigs.

Parameters	Groups	BL	H30 min	T30 min	T60 min	T120 min	T180 min	T240 min	T300 min
**Arterial blood pressure**	**control**	87±5	30±2#	84±5	101±7	106±15	110±11	110±17	105±19
**(mmHg)**	**sabiporide**	90±6	33±1#	103±4# *	110±5#	116±3#	106±6#	111±9#	99±9
**Heart rate**	**control**	66±5	125±12#	97±11	99±12	120±26#	107±28	110±13	122±20#
**(beats/min)**	**sabiporide**	70±5	141±13#	95±9	110±10#	114±10#	115±19#	115±13#	123±10#
**Right atrial pressure**	**control**	8.1±0.8	4.9±0.5	19.5±1.8#	24.5±3.4#	24.4±5.6#	16.4±3.6#	17.5±3.5#	16.9±3.4#
**(mmHg)**	**sabiporide**	10.2±1.0	6.1±0.7	21.6±3.9#	23.2±5.7#	24.2±4.2#	15.8±2.0	12.9±1.6*	12.0±1.5*
**Systemic vascular resistance**	**control**	65±5	43±5#	58±7	57±10	66±15	87±20	94±19	98±21#
**(mmHg mL^−1^ kg^−1^ min^−1^)**	**sabiporide**	59±4	40±7#	76±14*	81±3*	74±8	85±11#	83±14#	74±14*
**Coronary perfusion pressure**	**control**	79±4	23±2#	70±6	76±7	81±18	94±14	92±19	88±20
**(mmHg)**	**sabiporide**	79±7	25±1#	82±3	87±3	91±5	97±9#	96±13#	87±9
**Cardiac output/body weight**	**control**	98±5	38±4#	103±17	116±18	98±7	89±9	79±2	71±3
**(mL^−1^ kg^−1^ min^−1^)**	**sabiporide**	105±6	43±14#	93±15#	95±5*	106±9	103±13	106±8*	107±15*

All values are the mean ± SD (n = 5–6).

* p<0.05 vs. the control group;

# p<0.05 vs. the baseline value. BL: baseline; H30: at 30 minutes of hypovolemia; T30 min, –T300 min: at 30 min, –and 300 min from lactic acid infusion.

Echocardiographic measurement shows that hypovolemia followed by a lactic acid infusion reduced left ventricular ejection fraction, decreased fractional shortening, and impaired wall motion (Table 3). In contrast, in animals given sabiporide left ventricular ejection fraction and fractional shortening were higher and wall motion was improved compared to vehicle control animals.

**Table 3:** Echocardiographic measurements of left ventricular ejection fraction, fractional shortening and wall motion score index.

	BL	H30 min	T120 min	T180 min	T300 min
**Ejection fraction (%)**					
Control	60.2±4.4	45.5±5.1#	35.4±3.9#	34.9±4.3#	31.2±3.6#
sabiporide	58.7±4.5	43.8±3.9#	46.2±4.1# *	48.2±4.1# *	42.7±3.7# *
**Fractional shortening (%)**					
Control	31.7±2.6	24.3±2.1#	15.3±2.5#	15.6±2.7#	11.8±2.4#
sabiporide	32.5±3.0	25.4±3.9#	21.6±3.1# *	23.3±2.9# *	20.4±2.8# *
**Wall Motion Score Index**					
Control	1.0±0.0	1.2±0.10	2.2±0.19#	2.5±0.17#	2.6±0.21#
sabiporide	1.0±0.0	1.2±0.11	1.5±0.17# *	1.7±0.13# *	1.8±0.15# *

All values are the mean ± SD (n = 5–6).

* p<0.05 vs. the control group;

# p<0.05 vs. the baseline value. BL: baseline; H30 min: end of hypovolemia; T120 min: end of lactic acid infusion; T180 min: end of fluid infusion; and T300 min: end of the experiment.

### Effects of Sabiporide on Blood Oxygenation

**Figure 4:**
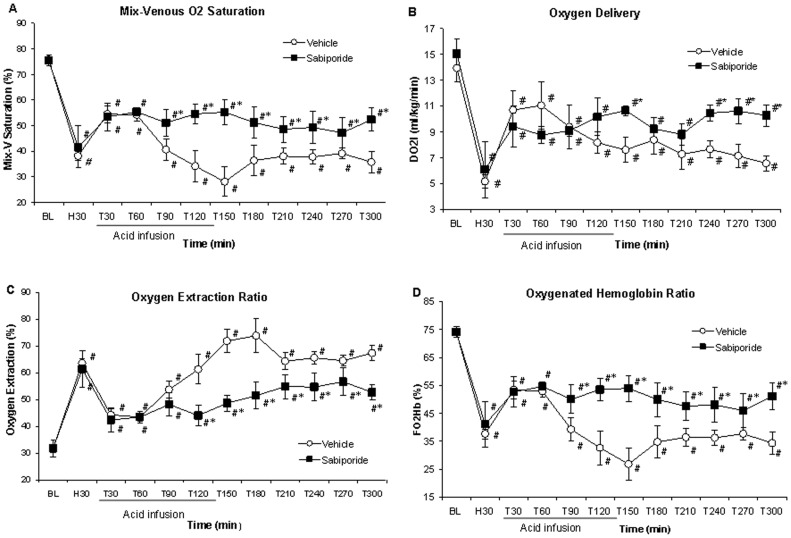
Changes in mixed-venous blood oxygen saturation, oxygen delivery, oxygen extraction ratio and mixed-venous blood oxygenated hemoglobin/total Hb ratio (FO_2_Hb) ratio. NHE1 inhibition with sabiporide prevented excessive fall in mixed-venous blood oxygen saturation following hypovolemia and lactic acidosis, and improved mixed-venous blood oxygen binding capacity of hemoglobin, resulting in improved oxygen delivery and decreased oxygen extraction ratio, suggesting improved tissue oxygenation. All values are the mean ± SD. N = 5–6. *p<0.05 vs. the control group; #p<0.05 vs. the baseline value.

As indicated in Table 1 and Figures 4A–D, there was a marked decrease in mixed venous blood oxygen saturation and oxygen content, associated with a marked increase in tissue oxygen extraction following hypovolemia in controls. There was no significant difference between two study groups. There was a temporary increase in mixed venous blood oxygen saturation and oxygenated hemoglobin ratio immediately after lactic acid infusion followed by sustained decrease throughout the experiment. Lactic acid infusion caused an initial return in oxygen delivery, but then followed by a decrease. Oxygen delivery continued to fall even after the acid infusion was terminated in control animals. Oxygen consumption in controls was increased during the acid infusion and remained above baseline level throughout the experiment (no significant difference vs. the baseline). Thus, in control animals, the imbalance between oxygen delivery and oxygen consumption resulted in increased oxygen extraction ratio. Oxygen extraction ratio reached 74% after lactic acid infusion, and remained high throughout the experiment, indicating low tissue oxygenation in control animals.

In contrast, in animals received sabiporide, the oxygen consumption did not increase to the same extend as in the control animals at the beginning of the acid infusion, but gradually increased reaching values 32% greater than the control group at the end of the experimental period. Sabiporide prevented the excessive fall in mixed-venous oxygen saturation and oxygen binding capacity of hemoglobin, resulting in improved oxygen delivery and decreased oxygen extraction ratio, consistent with improved tissue oxygenation.

### Effects of Sabiporide on Inflammatory Response and Indices of Organ Injury

The hypotension and infusion of lactic acid resulted in a significant increase in proinflammatory cytokine production in controls (Table 4). In the animals receiving sabiporide, the increase in TNF-α and IL-6 levels noted in controls were reduced by 50% and 63%, respectively.

**Table 4:** Changes of plasma levels of TNF-α, IL-6, troponin-I, ALT, AST and urea following severe lactic acidosis in pigs.

	BL	H30 min	T120 min	T300 min
**TNF-α (pg/ml)**				
Control	170.7±51.1	195.6±56.3	327.0±75.2#	579.9±93.1#
sabiporide	178.2±46.2	201.9±51.2	210.5±52.3*	288.4±32.1# *
**IL-6 (pg/ml)**				
Control	729±146	795±176	1312±201#	2813±355#
sabiporide	714±128	774±143	877±151	1037±220# *
**Troponin-I (ng/ml)**				
Control	0.23±0.03	0.29±0.08	7.82±1.21#	97.7±16.2#
sabiporide	0.26±0.02	0.27±0.05	5.31±0.82#	45.3±7.9# *
**ALT (U/L)**				
Control	13.7±2.7	15.6±3.4	30.1±4.9#	48.3±6.2#
sabiporide	14.1±3.9	14.9±3.3	19.5±3.1*	31.7±4.0# *
**AST (U/L)**				
Control	92.3±14.2	101.5±20.2	204.7±30.1#	327.5±38.8#
sabiporide	87.4±16.3	94.1±26.2	137.2±25.9# *	211.3±27.5# *
**Urea (mg/dl)**				
Control	57.2±6.5	63.4±8.2	128.1±21.2#	235.0±30.4#
sabiporide	61.4±7.8	65.9±9.5	89.4±11.8# *	140.3±24.8# *

All values are the mean ± SD (n = 5–6).

* p<0.05 vs. the control group;

# p<0.05 vs. the baseline value. BL: baseline; H30: at 30 minutes of hypovolemia; T120 min: end of lactic acid infusion; and T300 min: end of the experiment.

Troponin-I levels in plasma were significantly elevated in controls following lactic acid infusion consistent with myocardial damage. The administration of sabiporide led to a 54% reduction in troponin-I levels compared to controls, indicating a reduction in myocardial injury. Similarly, plasma levels of ALT and AST (markers of liver injury) were also significantly increased in controls. However, in animals treated with sabiporide, the increase in ALT level was reduced by 34%, and AST level was reduced by 35% compared to controls. We measured the rise in plasma levels of urea (an indicator of impaired excretory function of the kidney and/or increased catabolism). Plasma levels of urea were significantly increased following severe lactic acidosis, and sabiporide treatment reduced urea levels by 40% compared to control animals.

## Discussion

Metabolic acidosis is common in seriously ill patients and its presence can have a deleterious impact on clinical outcome [1]–[3]. Administration of base, a common therapeutic maneuver does not appreciably improve clinical outcome, even when acidemia is improved [24]. The results of the present study demonstrate that administration of the potent NHE1 selective inhibitor, sabiporide to animals with metabolic acidosis induced by hypoperfusion and a lactic acid infusion, improved cardiac function and the delivery of oxygen, and reduced the excessive increase in pulmonary artery pressure and pulmonary vascular resistance observed in controls. Administration of this NHE1 inhibitor also reduced the proinflammatory cytokine production, lessened the severity of myocardial and liver damage, and reduced mortality.

Several different models have been used experimentally to simulate lactic acidosis in humans. Infusion of lactic acid to produce metabolic acidosis, although it does not duplicate all the cellular changes observed with lactic acidosis observed clinically, it is associated with depression of cardiac contractility [20], [21]. Therefore, it seems a reasonable model to examine the potential benefits of inhibition of NHE1.

The improvement in cardiovascular function produced by administration of a NHE1 inhibitor is similar to that found in studies of severe hemorrhagic shock reported by our group previously [17], [25]. It is also consistent with a separate study in rats with sepsis given amiloride at doses designed to block NHE1 [18]. In our recent study in a porcine model of asphyxia-induced cardiac arrest, a model characterized by hypoxia and the resultant global lactic acidosis, we showed that post-arrest administration of sabiporide (3 mg/kg) attenuated cardio-pulmonary dysfunction, improving regional blood flows to vital organs (brain, heart, kidney, liver, spleen, etc.), resulting in reduced pro-inflammatory response [19]. In the present study, treatment with sabiporide also attenuated the acidosis-induced excessive increase in pulmonary arterial pressure and pulmonary vascular resistance, improved hemoglobin-oxygen binding capacity, and mixed-venous blood oxygen saturation. The evidence of less myocardial and liver damage is also consistent with improved tissue perfusion resulting from s treatment.

Mortality in patients with metabolic acidosis is increased and with lactic acidosis can reach values of 60 to 80% [26]. Therefore, evidence that therapy can reduce mortality as observed in the present study is important. The reduction in mortality with the NHE1 inhibitor reported in the present study mirrored the reduction in mortality observed by our group in animals subject to severe hemorrhage (50 ml/kg) treated with an NHE1 inhibitor [17]. These early deaths were due to myocardial arrhythmias and fibrillation-induced sudden cardiac arrest. NHE1 inhibitors protect from myocardial ischemia-reperfusion induced arrhythmias and fibrillation has been previously reported [14]–[16], [27]. Our recent studies showed that in a rat model of regional myocardial ischemia-reperfusion injury, sabiporide reduced ischemia-induced ventricular arrhythmias and completed prevented fibrillation-induced early death [28]. Thus, it is not surprising that sabiporide prevented arrhythmias and fibrillation-induced early death in the present study.

In addition to impaired cardiovascular function, severe metabolic acidosis has been shown to stimulate an inflammatory state and possibly alter the immune response [7], [26]. In this regard, macrophage production of interleukins is stimulated and lymphocyte function is suppressed with metabolic acidosis, leading to increased inflammation and an impaired immune response [26]. Furthermore, extracellular acidification has been shown to induce human neutrophil activation [29] and acidosis induces CD18 mediated neutrophil–endothelial adhesion and may lead to vascular dysfunction [30]. In previous studies, NHE1 inhibition has been shown to reduce neutrophil accumulation, chemokine production and NF-κB activation, and attenuate leukocyte-endothelial cell interactions, suggesting a possible pathological role of activation of NHE1 in production of tissue inflammatory injury in various experimental settings [13], [25], [31]. Our recent study in a pig model of traumatic hemorrhagic shock, we also showed NHE1 inhibition inhibits NF-κB activation and neutrophil infiltration, reduces iNOS expression and ERK1/2 phosphorylation, thereby, reducing systemic inflammation and thus multi-organ injury [32]. In the present study, hypotension and lactic acid infusion resulted in increased proinflammatory cytokine production, whereas, NHE1 inhibition lessened the production of proinflammatory cytokines TNF-α and IL-6. These findings suggest that activation of NHE1 might mediate the inflammatory response found with acute metabolic acidosis.

Excellent reviews have been published on the pathogenic mechanisms of pH-regulatory NHE1 activation and protective actions of NHE1 inhibitors via attenuation of cellular ionic derangement [14]–[16]. The application of these concepts to the settings of systemic metabolic acidosis is novel. Although most studies investigated the protective effects of NHE1 inhibition in cardiac myocytes/heart, the fact is that NHE1 is ubiquitously expressed in all mammalian cells [14]–[16]. Thus, our demonstration of whole body protective actions by a potent selective NHE1 inhibitor in a setting of global metabolic acidosis in vivo should lead to a refocus of the therapeutic potential of NHE1 inhibitors: a whole body protection from systemic metabolic acidosis.

Taken as a whole, these studies indicate that treatment with sabiporide can improve cardiovascular performance in organisms with acute metabolic acidosis, lessen the inflammatory response, prevent or lessen tissue injury, and reduce mortality. Given, the lack of effective therapies for the treatment of acute metabolic acidosis, these observations could support the utilization of administration of NHE1 inhibitor as an ancillary measure to efforts designed to improve cardiovascular and metabolic function in patients with acute severe metabolic acidosis.

## References

[1] Gunnerson KJ, Saul M, He S, Kellum JA (2006) Lactate versus non-lactate metabolic acidosis: a retrospective outcome evaluation of critically ill patients. Crit Care 10: R22. 16507145 10.1186/cc3987 PMC1550830

[2] Siesjo BK, Katsura KI, Kristian T, Li PA, Siesjo P (1996) Molecular mechanisms of acidosis-mediated damage. Acta Neurochir Suppl 66: 8–14. 8780790 10.1007/978-3-7091-9465-2_2

[3] Kraut JA, Madias NE (2010) Metabolic acidosis: pathophysiology, diagnosis and management. Nat Rev Nephrol 6: 274–285. 20308999 10.1038/nrneph.2010.33

[4] Marsiglia JC, Cingolani HE, Gonzalez N (1973) Relevance of beta receptor blockade to the negative inotropic effect induced by metabolic acidosis. Cardiovasc Res 7: 336–343. 4722931 10.1093/cvr/7.3.336

[5] Cingolani HE, Faulkner SL, Mattiazzi AR, Bender HW, Graham TP Jr (1975) Depression of human myocardial contractility with “respiratory” and “metabolic” acidosis. Surgery 77: 427–432. 1124498

[6] Brimioulle S, Lejeune P, Vachiery JL, Leeman M, Melot C, et al (1990) Effects of acidosis and alkalosis on hypoxic pulmonary vasoconstriction in dogs. Am J Physiol 258: H347–H353. 2309902 10.1152/ajpheart.1990.258.2.H347

[7] Kellum JA, Song M, Li J (2001) Lactic and hydrochloric acids induce different patterns of inflammatory response in LPS-stimulated RAW 264.7 cells. Am J Physiol Regul Integr Comp Physiol 286: R686–692. 10.1152/ajpregu.00564.2003 14695114

[8] Kellum JA, Song M, Venkataraman R (2004) Effects of hyperchloremic acidosis on arterial pressure and circulating inflammatory molecules in experimental sepsis. Chest 125: 243–248. 14718447 10.1378/chest.125.1.243

[9] Webster KA, Discher DJ, Kaiser S, Hernandez O, Sato B, et al (1999) Hypoxia-activated apoptosis of cardiac myocytes requires reoxygenation or a pH shift and is independent of p53. J Clin Invest 104: 239–252. 10430605 10.1172/JCI5871 PMC408414

[10] Adrogue HJ, Madias NE (1998) Management of life-threatening acid-base disorders. Second of two parts. N Engl J Med 338: 107–111. 9420343 10.1056/NEJM199801083380207

[11] Mathieu D, Neviere R, Billard V, Fleyfel M, Wattel F (1991) Effects of bicarbonate therapy on hemodynamics and tissue oxygenation in patients with lactic acidosis: a prospective, controlled clinical study. Crit Care Med 19: 1352–1356. 1935152 10.1097/00003246-199111000-00008

[12] Huseby JS, Gumprecht DG (1981) Hemodynamic effects of rapid bolus hypertonic sodium bicarbonate. Chest 79: 552–554. 7226933 10.1378/chest.79.5.552

[13] Forsythe SM, Schmidt GA (2000) Sodium bicarbonate for the treatment of lactic acidosis. Chest 117: 260–267. 10631227 10.1378/chest.117.1.260

[14] Anderson SE, Murphy E, Steenbergen C, London RE, Cala PM (1990) Na^+^/H^+^ exchange in myocardium: effects of hypoxia and acidification on Na and Ca. Am J Physiol 259: C940–C948. 2175547 10.1152/ajpcell.1990.259.6.C940

[15] Vaughan-Jones RD, Villafuerte FC, Swietach P, Yamamoto T, Rossini A, et al (2006) pH-Regulated Na(+) influx into the mammalian ventricular myocyte: the relative role of Na(+)-H(+) exchange and Na(+)-HCO Co-transport. J Cardiovasc Electrophysiol 17: S134–S140. 16686668 10.1111/j.1540-8167.2006.00394.x

[16] Karmazyn M (1999) Mechanisms of protection of the ischemic and reperfused myocardium by sodium-hydrogen exchange inhibition. J Thromb Thrombolysis 8: 33–38. 10481212 10.1023/a:1008990530176

[17] Wu D, Arias J, Bassuk J, Doods H, Seidler R, et al (2008) Na+/H+ exchange inhibition delays the onset of hypovolemic circulatory shock in pigs. Shock 29: 519–525. 17724433 10.1097/shk.0b013e318150757a

[18] Sikes PJ, Zhao P, Maass DL, White J, Horton JW (2005) Sodium/hydrogen exchange activity in sepsis and in sepsis complicated by previous injury: 31P and 23Na NMR study. Crit Care Med 33: 605–615. 15753754 10.1097/01.ccm.0000155910.89252.fe

[19] Lin X, Lee D, Wu D (2012) Protective effects of NHE1 inhibition with sabiporide in an experimental model of asphyxia-induced cardiac arrest in piglets. Resuscitation. Sep 15. Epub ahead of print. 10.1016/j.resuscitation.2012.08.334 22989728

[20] Cooper DJ, Herbertson MJ, Werner HA, Walley KR (1993) Bicarbonate does not increase left ventricular contractility during L-lactic acidemia in pigs. Am Rev Respir Dis 148: 317–322. 8342893 10.1164/ajrccm/148.2.317

[21] Wiklund L, Sahlin K (1985) Induction and treatment of metabolic acidosis: a study of pH changes in porcine skeletal muscle and cerebrospinal fluid. Crit Care Med 13: 109–113. 3917889 10.1097/00003246-198502000-00012

[22] Sahn DJ, DeMaria A, Kisslo J, Weyman A (1978) Recommendations regarding quantitation in M-mode echocardiography: results of a survey of echocardiographic measurements. Circulation. 58: 1072–1083. 10.1161/01.cir.58.6.1072 709763

[23] Schiller NB, Shah PM, Crawford M, DeMaria A, Devereux R, et al (1989) Recommendations for quantitation of the left ventricle by two-dimensional echocardiography. American Society of Echocardiography Committee on Standards, Subcommittee on Quantitation of Two-Dimensional Echocardiograms. J Am Soc Echocardiogr. 2: 358–367. 10.1016/s0894-7317(89)80014-8 2698218

[24] Kraut JA, Kurtz I (2005) Metabolic acidosis of CKD: diagnosis, clinical characteristics, and treatment. Am J Kidney Dis 45: 978–993. 15957126 10.1053/j.ajkd.2005.03.003

[25] Wu D, Dai H, Arias J, Latta L, Abraham WM (2009) Low-volume resuscitation from traumatic hemorrhagic shock with Na+/H+ exchanger inhibitor. Crit Care Med 37: 1994–1999. 19384202 10.1097/CCM.0b013e3181a0052e

[26] Kellum JA, Song M, Li J (2004) Science review: extracellular acidosis and the immune response: clinical and physiologic implications. Crit Care 8: 331–336. 15469594 10.1186/cc2900 PMC1065014

[27] Wu D, Stassen JM, Seidler R, Doods H (2000) Effects of BIIB513 on ischemia-induced arrhythmias and myocardial infarction in anesthetized rats. Basic Res Cardiol. 95: 449–456. 10.1007/s003950070020 11192365

[28] Doods H, Wu D (2013) Sabiporide reduces ischemia-induced arrhythmias and myocardial infarction, attenuates ERK phosphorylation and iNOS induction in rats. Journal of Biomedicine and Biotechnology, in press. 10.1155/2013/504320 PMC3591136 23484128

[29] Martínez D, Vermeulen M, Trevani A, Ceballos A, Sabatté J, et al (2006) Extracellular acidosis induces neutrophil activation by a mechanism dependent on activation of phosphatidylinositol 3-kinase/Akt and ERK pathways. J Immunol 176: 1163–1171. 16394005 10.4049/jimmunol.176.2.1163

[30] Serrano CV Jr, Fraticelli A, Paniccia R, Teti A, Noble B, et al (1996) pH dependence of neutrophil-endothelial cell adhesion and adhesion molecule expression. Am J Physiol 271: C962–C970. 8843727 10.1152/ajpcell.1996.271.3.C962

[31] Nemeth ZH, Deitch EA, Lu Q, Szabo C, Hasko G (2002) NHE blockade inhibits chemokine production and NF-kappaB activation in immunostimulated endothelial cells. Am J Physiol Cell Physiol 283: C396–C403. 12107048 10.1152/ajpcell.00491.2001

[32] Wu D, Qi J (2012) Mechanisms of the beneficial effect of NHE1 inhibitor in traumatic hemorrhage: Inhibition of inflammatory pathways. Resuscitation 83: 774–781. 22155220 10.1016/j.resuscitation.2011.11.025

